# The mediating role of psychological factors in the relationship between body image dissatisfaction and eating disorders: a cross-sectional study in adults

**DOI:** 10.3389/fnut.2026.1881290

**Published:** 2026-08-24

**Authors:** Nazlı Nur Aslan Çin, Murat Açık, Lina Begdache

**Affiliations:** 1 Department of Nutrition and Dietetics, Faculty of Health Sciences, Karadeniz Technical University, Trabzon, Türkiye; 2 Department of Nutrition and Dietetics, Faculty of Health Sciences, Fırat University, Elâzığ, Türkiye; 3 Department of Health and Wellness Studies, Decker College of Nursing and Health Sciences, Binghamton University, State University of New York, Binghamton, NY, United States

**Keywords:** anxiety, body image disturbance, depression, eating disorders, guilt, mediation analysis, mental health, nutrition

## Abstract

**Background/objectives:**

Body image disturbance is a well-established risk factor for eating disorders; however, the psychological mechanisms through which it exerts its influence remain insufficiently understood. This study examined the mediating roles of guilt, shame, anxiety, depression, and distress in the relationship between body image disturbance and eating disorder behaviors in adults.

**Methods:**

This cross-sectional study included 1,576 adults aged 19–64 years from Elazığ province, Turkey. Participants completed the Body Image Disturbance Questionnaire (BIDQ), Eating Disorder Examination Questionnaire short form (EDE-Q-13), Guilt and Shame Proneness Scale (GASP), and Depression Anxiety Stress Scale-21 (DASS-21). Hierarchical regression and path analyses were conducted to identify predictors and mediators of eating disorder symptoms, with gender, location, and BMI as covariates.

**Results:**

Body image disturbance was the strongest independent predictor of eating disorder symptoms, explaining 24% of the variance (*β* = 0.89). Higher symptom scores were significantly associated with elevated depression, anxiety, and distress levels, with moderate-to-large effect sizes. Path analyses demonstrated that guilt oriented toward negative behavioral evaluation (Guilt-NBE), shame-related withdrawal, and depression partially mediated the BIDQ–EDE-Q relationship, with the depression model accounting for 28.3% of total variance. Anxiety similarly mediated this relationship (28.3%), whereas distress explained a slightly lower proportion (27.3%).

**Conclusion:**

Body image disturbance was statistically associated with eating pathology, with depression, anxiety, and shame-based withdrawal emerging as significant indirect pathways in the mediation model, while behaviorally focused guilt demonstrated a pattern consistent with a self-regulatory function. Interventions targeting maladaptive shame and affective distress, alongside strategies supporting adaptive guilt processing, may constitute meaningful clinical targets in eating disorder prevention and treatment.

## 1 Introduction

Eating disorders are fatal psychiatric conditions characterized by substantial and enduring impairments in eating patterns, negatively impacting physical health, psychological well-being, and social quality of life (1). These disorders, comprising subtypes such as anorexia nervosa, bulimia nervosa, and binge eating disorder, are particularly prevalent among young women in early adulthood. Research conducted on a global scale indicates that between 5.5% and 17.9% of young women and 0.6% to 2.4% of young males suffer from eating disorders that satisfy the diagnostic standard of the DSM-5. However, these prevalence rates vary considerably by age, gender, and geographic region (2).

Research indicates that body image plays a significant role in the development of disordered eating behaviors and is a key factor in both diagnosis and treatment (3). Indeed, the cultivation of a positive body image is often regarded as a prerequisite for full recovery (4). Body image is a multidimensional construct encompassing an individual’s internal representation of their weight, shape, and physical appearance. It includes cognitive-emotional, perceptual, and behavioral dimensions (5). The perceptual component refers to distortions in the way one’s body is visually perceived, while the cognitive-emotional dimension involves thoughts and feelings about the body shape, particularly body dissatisfaction, which are known to contribute significantly to the onset of eating disorders (6). As highlighted by Mallaram et al. (3), unrealistic and negative evaluations of one’s physical appearance can induce profound dissatisfaction, which subsequently triggers disordered eating patterns.

This phenomenon is particularly noticeable during adolescence and early adulthood, periods heavily influenced by societal beauty standards and the pervasive influence of social media (7). The dissemination of perfectionist and often unattainable ideals, exacerbated during periods of heightened uncertainty and social isolation, particularly post-pandemic era, has intensified body dissatisfaction among young adults (7). Such dissatisfaction often leads to maladaptive behaviors, including excessive dieting, food restriction, or emotional eating (8, 9). These acts are frequently associated with strong negative emotions like guilt and shame. For example, the person may feel guilt losing self-control, while fear of being judged by societal judgment may cause humiliation (10). This emotional burden not only facilitates the development of eating disorders but also contributes to their chronicity (11).

During the COVID-19 epidemic, social isolation and heightened uncertainty contributed to a negative body image perception and an upsurge in eating disorders (12). When individuals perceive a failure to meet internalized body standards, guilt may arise, whereas shame may develop from the fear of social rejection (13). Research shows that guilt and shame are key emotional mediators influencing the relationship between body image disturbance and eating disorders (14, 15). These emotions can impair adaptive coping mechanisms, leading to the persistence of maladaptive eating habits (16, 17). Furthermore, self-conscious emotions like shame and guilt can negatively impact cognitive flexibility and compromise emotion-regulation abilities (18, 19). Additional psychological factors, including depression and anxiety, significantly contribute to the onset and persistence of eating disorders. Anxiety may disrupt eating patterns through persistent tension, resulting in restrictive behaviors driven by a desire for control or, conversely, stress-induced binge eating (6, 20). Conversely, depression is linked to diminished energy, despair, and a reduced feeling of self-esteem, which heightens the likelihood of eating disorders by perpetuating negative body images (21). It is posited that depression and anxiety interact synergistically with guilt and shame, further entrenching the chronicity of these disorders (22).

Importantly, the existing evidence identifying guilt, shame, depression, and anxiety as mediators is derived predominantly from Western, individualistic societies. For many years, eating disorders were conceptualised as culture-bound conditions specific to affluent Western populations (23). This view has since been challenged, as disordered eating is now increasing in many non-Western and rapidly modernising societies, driven by urbanisation and the global spread of the Western “thin ideal” through mass and social media (23, 24). At the same time, it cannot be assumed that these emotional mechanisms operate uniformly across cultures; indeed, some mediating pathways identified in Western samples have not been replicated in other ethnic or cultural groups (25). Turkey provides a particularly informative context in which to examine this issue, as it lies at the intersection of Eastern and Western influences, where rapid urbanisation and high social media use coexist with collectivist values in which social reputation, family honour, and the evaluation of others strongly shape self-worth (26, 27). Such cultural characteristics may be especially relevant to shame, a fundamentally social emotion rooted in the fear of negative evaluation and devaluation by others. Although recent studies suggest that body image concerns and disordered eating in Turkey are approaching levels observed in Western populations (26, 28), the emotional mechanisms linking body image disturbance to disordered eating have rarely been examined in this population. Examining these pathways in a large Turkish adult sample therefore provides an opportunity to assess the extent to which current theoretical models generalise beyond Western contexts.

Although previous research has largely examined the independent effects of body dissatisfaction, guilt, shame, anxiety, and depression on eating disorders (12, 25), their combined and indirect roles have been investigated predominantly in Western populations and remain underexplored in non-Western contexts (24, 26). The present study therefore examined whether the established mediating roles of guilt, shame, anxiety, and depression in the relationship between body image disturbance and eating disorders generalise to a non-Western adult population, using a large community-based sample from Turkey. By testing these pathways outside the Western settings in which they were originally established, this study aimed to determine whether these mechanisms are culturally robust or context specific.

## 2 Materials and methods

This cross-sectional study was conducted in the Department of Nutrition and Dietetics, Fırat University, from April to July 2023. Participants between the ages of 19–64 living in Elazığ province were contacted on social media platforms and invited to participate in the research study. A Snowball Sampling method was used to increase the sample size by asking participants to share the survey link with two or more adult friends. The participants invited to the study were initially evaluated against the inclusion and exclusion criteria. Informed consent was presented to participants on the first page before accessing the survey. Each participant who met the study criteria was included after reading and signing the informed consent form. Consequently, the researchers administered a short sociodemographic questionnaire (age, marital status, current weight, etc.) and the self-report instruments described below to participants (total response time of approximately 30 min). Participants did not receive any incentive or compensation for their time. The study was conducted in accordance with the principles of the Declaration of Helsinki and was approved by the Ethics Committee of Firat University.

The eligibility criteria to participate in the study were: (1) 19–64 years, (2) native speaker of the Turkish language. Exclusion criteria of the study: (1) if they had a BMI indicative of severe underweight (≤16.50 kg/m^2^) or morbid obesity (≥45.00 kg/m^2^), (2) those diagnosed with at least three comorbid diseases, (3) the presence of psychiatric or neurological diagnosis, (4) not receiving any nutritional therapy. These exclusion criteria were applied to ensure a community-based sample of generally healthy adults, thereby minimising the confounding influence of severe metabolic conditions, significant medical burden, and pre-existing psychiatric or neurological diagnoses on self-reported eating behaviors and emotional regulation patterns. After applying these criteria, a final sample of 1,576 participants was retained for analysis. Participants with missing data on any study variable were excluded prior to the application of eligibility criteria via listwise deletion.

### 2.1 Instruments and measures

The research protocol included sociodemographic questions (e.g., sex, age, educational level, marital status, area of residence, physical activity levels) and clinical information (e.g., current psychological or psychiatric support). Participants also completed the Turkish validated versions of the following self-report instruments. Body mass index (BMI) was calculated using the Quetelet method (kg/m^2^), based on self-reported current weight (kilograms) and height (meters) of participants.

### 2.2 Eating disorder examination questionnaire (EDE-Q-13)

The EDE-Q developed by Fairburn and Beglin was developed to assess eating disorders and consists of 28 items. Levari et al. conducted a validity and reliability study of the short version of the EDE-Q. This scale showed a strong positive correlation with the original EDE-Q. The EDE-Q short form includes questions to examine the eating behaviors of the individuals for the last 4 weeks (28 days). EDE-Q consists of 13 items and 5 subscales, namely Eating Restraint (ER), Shape and Weight Over-Evaluation (SWO), Body Dissatisfaction (BD), Bingeing, and Purging. Each item in EDE-Q is scored on a 7-point Likert-type scale (0 = never, 1 = 1–5 days, 2 = 6–12 days, 3 = 13–15 days, 4 = 16–22 days, 5 = 23–27 days, 6 = every day). The scores of the scale are evaluated based on the sum of the scores of the items in the entire scale or each subscale of the scale, and higher scores indicate higher levels of eating-related psychopathology (29). The Turkish validity and reliability study of the EDE-Q short form was conducted by Esin and Ayyıldız, and the Cronbach’s alpha value of the scale was 0.89 and the values of the 5 subscales were calculated in the range of 0.75–0.94 (30).

### 2.3 Body image disturbance questionnaire (BIDQ)

This scale was developed by Cash et al. (31) based on the Body Dysmorphic Disorder Questionnaire designed by Phillips in 1996. The BIDQ, which was developed to assess body image disorder, consists of 7 items; (1) concern about some part(s) of the body felt to be unattractive (rated from 1 = “not at all concerned” to 5 = “extremely concerned”); (2) mental preoccupation with these concerns (rated from 1 = “not at all preoccupied” to 5 = “extremely preoccupied”); (3) experiences of emotional distress over the “defect” (rated from 1 = “no distress” to 5 = “extreme and disabling”); (4) its production of impairment in social, occupational, or other important areas of functioning (rated from 1 = “no limitation” to 5 = “extreme, incapacitating”); (5) its interference with social life (rated from 1 = “never” to 5 = “very often”); (6) interference with school, job, or role functioning (rated from 1 = “never” to 5 = “very often”); (7) avoidance of things due to the “defect” (rated from 1 = “never” to 5 = “very often”). The scores obtained from the 7 questions of the scale ranges from 7 to 35. High scores on the questionnaire indicate negative body perception and impaired psychosocial functioning, while low scores indicate no problem with body image (31). The reliability and validity study of BIBA in Turkish was carried out by Kuzu et al. (32) and the internal consistency coefficient (Cronbach’s *α*) was calculated as 0.88.

### 2.4 Guilt and shame proneness scale (GASP)

The guilt and shame proneness scale, developed by Cohen et al. (33) is a 16-item scale, which measures one’s proneness to experience shame and guilt. Each item contains a transgression scenario together with a possible reaction that might be given in that situation (e.g., “You give a bad presentation at work. Afterwards your boss tells your coworkers it was your fault that your company lost the contract. What is the likelihood that you would feel incompetent?”). Respondents are expected to rate the likelihood of feeling, behaving or thinking in the stated way for each scenario on a 7-point scale, ranging from “very unlikely” to “very likely.” The scale consists of four subscales: Guilt-NBE (Negative Behavior Evaluations), Guilt-Repair, Shame-NSE (Negative Self Evaluations), Shame-Withdraw (33). A score for each subscale is calculated by taking the average of the four items related to that subscale. The GASP scale was verified in Turkey by Topçu (34).

### 2.5 Depression anxiety stress scale-21 (DASS-21)

The DASS-21 was developed by Lovibond and Lovibond (35) as a shortened version of the original 42-item scale. The scale consists of 21 items organized into three distinct sub-dimensions: Depression, Anxiety, and Stress, with each subscale containing seven items. To calculate the final score, the sum of the items for each subscale is multiplied by two. Higher scores indicate greater severity of the respective emotional distress, whereas lower scores lower scores indicate lower symptom levels. The Turkish adaptation, validity, and reliability study of the scale was conducted by Sarıçam (36) using both clinical and healthy control samples. The psychometric analysis demonstrated strong internal consistency; Cronbach’s alpha coefficients for the healthy control sample were calculated as 0.85 for depression, 0.80 for anxiety, and 0.77 for stress.

### 2.6 Statistical analysis

Data analyses were conducted using the JASP Statistical Software version of 0.18.2^1^. As there were no cut-off values for the EDE-Q scale, classification was based on the median (EDE-Q median cutoff ≤0.90 or >0.90). As the numerical data were scale scores, normality assumption was ensured by applying the z-score transformation. Thus, the normality assumption was provided for the hierarchical regression, path and t-test analysis. We performed t-test for comparisons of numerical variables between median groups and Pearson chi-square test for categorical data. In addition, Cohens’ effect size values were calculated to determine the effect sizes in the comparison of the quantitative data between the median groups. In case the Cohens’d effect size value is in the range of 0.2–0.5, 0.5–0.8 and greater than 0.8, it is shown as small, medium and large, respectively. The continuous variables were presented as mean ± standard deviation, and the categorical data were shown as proportion (N). Pearson correlations were used to the associations among BMI, EDE-Q, BIDQ, GASP, DASS-21 and their subscales. Pearson correlation coefficient, and its strength was classified according to r values as very strong (*r* = 0.80–1.00), strong (*r* = 0.60–0.79), moderate/modest (0.40–0.59), weak (0.20–0.39), or negligible (0.00–0.19). Hierarchical regression analysis was carried out to estimate the predictors of the EDE-Q. Based on the correlation matrix output, the scales or sub-dimensions of the scales found statistically significant with EDE-Q were treated as independent variables, and entered separately at each step. In addition, gender, location, and BMI were included as covariate. In each step, we presented the unstandardised beta coefficient (standard error), confidence intervals, coefficient of explanation (ΔR2), and ΔF-test score changes for the independent variables.

Path analysis was applied to assess the proposed theoretical models. At the first step, conceptual models were developed based on information obtained from literature. In the second step, a path analysis diagram was created according to the correlation matrix output. In the path analysis, whilst BIDQ is the independent variable, EDE-Q is positioned as a latent variable. Consistent with the variable-selection approach used in the hierarchical regression, only those GASP and DASS-21 sub-dimensions that were strongly linked with EDE-Q in the correlation matrix were inserted as mediating variables (37).

## 3 Results

### 3.1 General characteristics of participants

The general characteristics of the participants were compared based on the median EDE-Q score (Table 1). The mean EDE-Q score for the total sample was 1.21 ± 1.14, with a median of 0.90 [IQR: 0.23–1.86]. Participants were stratified into two groups based on the median cutoff (≤0.90 and >0.90). The distribution of age, education level, smoking status, and physical activity levels was similar between the two groups. However, the group with higher EDE-Q scores (>0.90) had a significantly higher proportion of women and a higher prevalence of chronic diseases compared to the lower-scoring group (*p* < 0.05). Additionally, participants living in metropolitan centers had significantly higher EDE-Q scores than those living in other locations. Alcohol consumption was also more frequent in the high EDE-Q group (>0.90) compared to non-consumers.

**Table 1:** Comparison of general characteristics according to EDE-Q median groups.

Variables	Total (*n* = 1,576)	EDE-Q ≤ 0.90 (*n* = 789)	EDE-Q > 0.90 (*n* = 787)	*p*
Age (yrs)	28.6 ± 9.5	28.3 ± 9.3	28.9 ± 9.7	0.225
Gender				0.003**
Male	608 (38.6%)	333 (54.8%)	275 (45.2%)	
Female	968 (61.4%)	456 (47.1%)	512 (52.9%)	
Education				0.076
Primary	59 (3.7%)	32 (54.2%)	27 (45.8%)	
High school	353 (22.4%)	161 (45.6%)	192 (54.4%)	
Associate degree	343 (21.8%)	162 (47.2%)	181 (52.8%)	
Undergraduate	821 (52.1%)	434 (52.9%)	387 (47.1%)	
Location				<0.001***
Township	357 (22.7%)	183 (51.3%)	174 (48.7%)	
City	525 (33.3%)	297 (56.6%)	228 (43.4%)	
Metropolitan	694 (44.0%)	309 (44.5%)	385 (55.5%)	
Smoking				0.062
Current	572 (36.3%)	284 (49.7%)	288 (50.3%)	
Never	978 (59.5%)	481 (51.3%)	457 (48.7%)	
Former	66 (4.2%)	24 (36.4%)	42 (63.6%)	
Alcohol				0.032*
No	1,378 (87.4%)	704 (51.1%)	674 (48.9%)	
Yes	198 (12.6%)	85 (42.9%)	113 (57.1%)	
Chronic disease				0.007**
No	1,340 (85.0%)	690 (51.5%)	650 (48.5%)	
Yes	236 (15.0%)	99 (41.9%)	137 (58.1%)	
Physical activity				0.241
Sedentary	1,221 (77.5%)	621 (50.9%)	600 (49.1%)	
Active	355 (22.5%)	168 (47.3%)	187 (52.7%)	

Categorical variables were analyzed using Pearson chi-square test; age was compared using an independent samples *t*-test. Data are presented as mean ± SD or *n* (%). *p < 0.05, **p < 0.01, ***p < 0.001. Bold values indicate statistically significant differences.

### 3.2 Comparison of BIDQ, GASP, mental health, and BMI between EDE-Q median groups

Table 2 presents the comparison of numerical variables between the EDE-Q median groups. BIDQ scores were statistically higher in individuals with elevated eating disorder symptoms (EDE-Q > 0.90), with a large effect size (Cohen’s d = −0.957). Similarly, mean levels of depression, anxiety, and stress were significantly higher in the EDE-Q > 0.90 group, with moderate effect sizes (Cohen’s d = −0.781, −0.777, and −0.639, respectively). Although BMI levels were higher in the group with elevated EDE-Q scores, the effect size was small. Regarding the GASP sub-dimensions, Guilt-NBE scores were relatively higher, while Shame-Withdraw scores were lower in the EDE-Q ≤ 0.90 group; however, these differences were not clinically significant.

**Table 2:** Comparison of BIDQ, GASP, mental health indicators, and BMI between EDE-Q median groups.

Variables	Total (*n* = 1,576)	EDE-Q ≤ 0.90 (*n* = 789)	EDE-Q > 0.90 (*n* = 787)	Cohen’s d	*p*
EDE-Q sub-scales					
ER	1.32 ± 1.54	0.40 ± 0.56	2.25 ± 1.56	−	−
SWO	1.51 ± 1.20	0.35 ± 0.24	2.78 ± 1.61	−	−
BD	1.80 ± 1.17	0.50 ± 0.31	3.12 ± 1.75	−	−
Bingeing	1.00 ± 0.83	0.23 ± 0.37	0.84 ± 0.61	−	−
Purging	0.45 ± 0.23	0.07 ± 0.20	0.84 ± 0.80	−	−
EDE-Q total	1.21 ± 0.90	0.30 ± 0.32	2.13 ± 0.70	−	−
BIDQ	1.61 ± 0.64	1.34 ± 0.42	1.89 ± 0.70	−0.957	<0.001***
GASP					
Guilt-NBE	5.25 ± 1.49	5.43 ± 1.52	5.06 ± 1.44	0.253	<0.001***
Guilt-repair	5.08 ± 1.41	5.14 ± 1.49	5.02 ± 1.33	0.083	0.100
Shame-NSE	4.89 ± 1.43	4.96 ± 1.48	4.87 ± 1.38	0.070	0.148
Shame-withdraw	3.83 ± 1.41	3.64 ± 1.47	4.01 ± 1.32	−0.265	<0.001***
Depression	6.09 ± 4.40	4.48 ± 3.70	7.71 ± 4.53	−0.781	<0.001***
Anxiety	4.60 ± 1.71	3.08 ± 1.36	6.12 ± 2.39	−0.777	<0.001***
Distress	7.24 ± 4.96	5.68 ± 4.60	8.80 ± 5.12	−0.639	<0.001***
BMI	23.3 ± 3.72	22.5 ± 2.95	24.2 ± 4.2	−0.451	<0.001***

Data were analyzed using an independent samples t-test. Cohen’s d values were calculated to evaluate effect size; small = 0.2, moderate = 0.5, large = 0.8. Data are presented as mean ± SD. BD, body dissatisfaction; BIDQ, body image disturbance questionnaire; BMI, body mass index; EDE-Q, eating disorder examination questionnaire; ER, eating restraint; GASP, guilt and shame proneness scale; Guilt-NBE, guilt-negative behavior evaluation; Shame-NSE, shame-negative self-evaluation; SWO, shape and weight over-evaluation. *p < 0.05, **p < 0.01, ***p < 0.001. Bold values indicate statistically significant differences.

### 3.3 Correlation analysis

**Figure 1:**
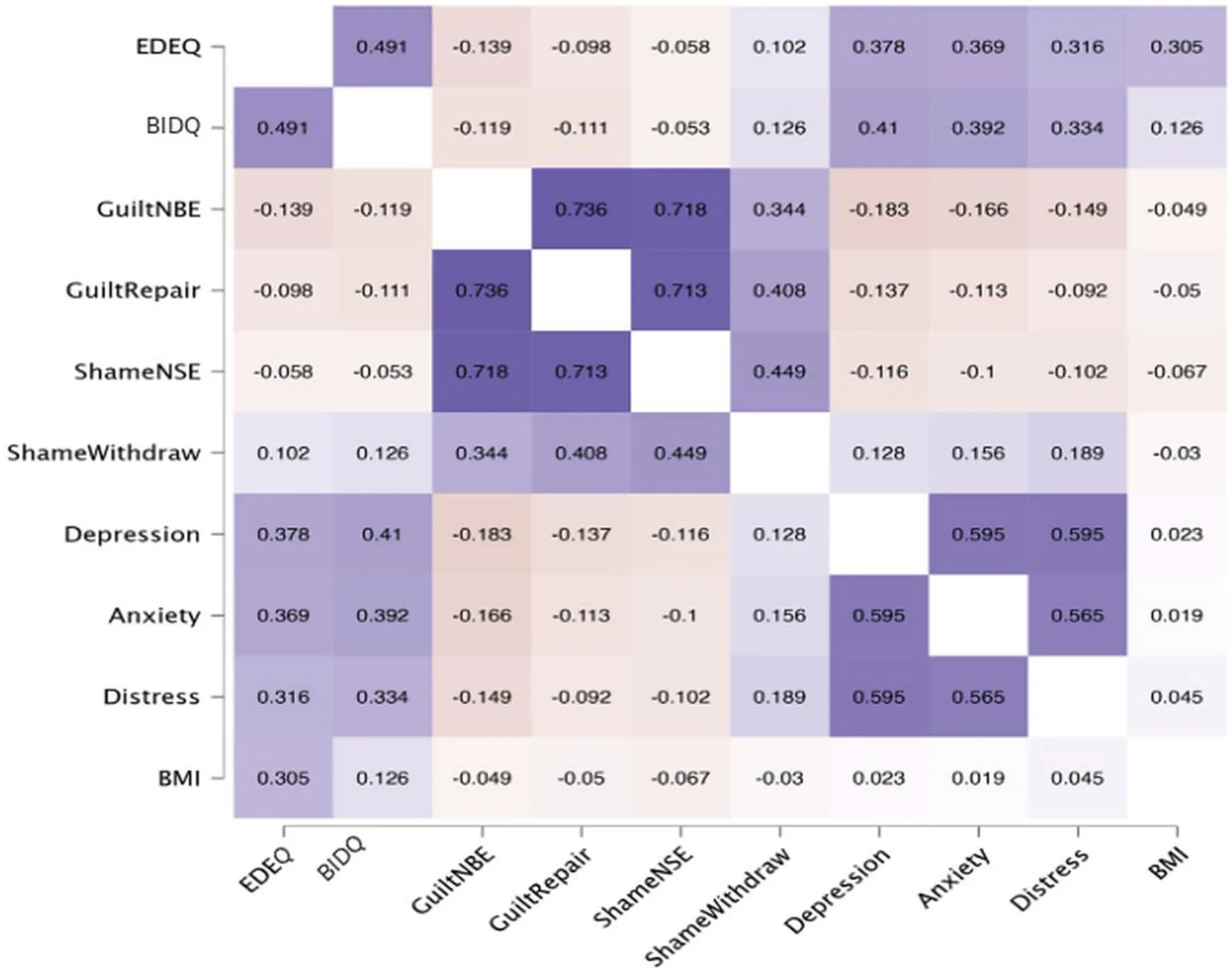
Pearson correlation heatmap matrix of EDE-Q, BIBA and components of GASP and mental disorder.

Figure 1 displays the correlations among EDE-Q, BIDQ, GASP, mental health indicators, and BMI. EDE-Q scores showed moderate positive correlations with BIDQ, depression, anxiety, stress, and BMI. However, EDE-Q was very weakly correlated with Guilt-NBE and shame-withdraw. BIDQ levels were moderately associated with depression, anxiety, and stress, but weakly associated with BMI. Guilt-NBE demonstrated negative associations with depression, anxiety, and stress, but a positive association with Shame-Withdraw.

### 3.4 Hierarchical regression analysis

Hierarchical regression analyses were conducted to identify predictors of EDE-Q scores based on the correlation matrix (Table 3). In the first step, BIDQ was identified as the strongest predictor of EDE-Q (*β*[SE] = 0.89 [0.06] and ΔF = 498.733), explaining 24% of the variance. BIDQ maintained its significant effect after adjusting for gender, location, and BMI. It was found to be statistically significant in the models established in the other two steps after fully adjusting (For step 2 and 3; ΔF and ΔR2: 14.105 and 0.012; 36.580 and 0.045, *p* < 0.001, respectively). Guilt-NBE (*β* = −0.13), depression (*β* = 0.03) and anxiety (*β* = 0.03) were statistically significant predictors of EDE-Q. In addition, it was found that all predictor variables explained 26.3% of EDE-Q in the model.

**Table 3:** Hierarchical regression model for eating behavior explained by body image disturbance, components of GASP, and mental health indicators.

Outcome variable	Predictor	Unadjusted model				Covariate-adjusted model^†^			
		*β* (SE)	95% CI	ΔF	Δ*R*^2^	*β* (SE)	95% CI	ΔF	Δ*R*^2^
Step 1	BIDQ	0.89 (0.06)	0.80–0.95	498.733	0.241	0.60 (0.04)	0.52–0.69	466.930	0.208
Step 2	Guilt-NBE	−0.13 (0.01)	−0.17–(−0.09)	12.827	0.012	−0.06 (0.01)	−0.02–(−0.11)	14.105	0.012
	Shame-withdraw	0.06 (0.01)	0.02–0.10			0.04 (0.01)	0.00–0.07		
Step 3	Depression	0.03 (0.00)	0.01–0.04	30.570	0.041	0.03 (0.00)	0.02–0.05	36.580	0.045
	Anxiety	0.03 (0.00)	0.02–0.05			0.03 (0.01)	0.02–0.05		

Unstandardised beta coefficients (SE) and 95% CI for independent variables were presented. In the final model, all independent variables included were statistically significant except for Shame-Withdraw in the covariate-adjusted model. ^†^Covariate-adjusted model controls for gender, location, and BMI.

Correlation analyses were performed between the sub-dimensions of the EDE-Q and BIDQ, GASP and mental disorder components (Supplementary Figure S1). According to the correlation matrix table, regression analyses were performed for the sub-dimensions of the EDE-Q (Table 4). After adjusting for covariates, BIDQ was positively associated with Eating Restraint (ER), Shape and Weight Over-evaluation (SWO), Body Dissatisfaction (BD), Bingeing, and Purging, explaining 9.1, 17.1, 18.4, 15.2, and 9.7% of the variance, respectively. Guilt-NBE was negatively associated with ER (*β*[SE] = −0.09[0.02]), Bingeing (*β*[SE] = −0.12[0.02]), and Purging (*β*[SE] = −0.13[0.02]). Distress was significantly associated only with BD. Depression and anxiety were positively associated with SWO and Purging. Independent predictors explained 18.5%, 19.5%, 20.9%, 23.3%, and 22.7% of ER, SWO, BD, bingeing and purging, respectively.

**Table 4:** Hierarchical regression model for EDE-Q subscales explained by BIDQ, GASP components, and mental health indicators.

Outcome variable	Predictor	*β* (SE)	95% CI	Δ*F*	Δ*R*^2^
ER					
Step 1	BIDQ	0.72 (0.05)	0.61–0.84	157.890	0.091
Step 2	Guilt-NBE	−0.09 (0.02)	−0.14–(−0.04)	24.999	0.025
Step 3	Anxiety	0.04 (0.01)	−0.01–0.03	38.653	0.069
SWO					
Step 1	BIDQ	1.13 (0.06)	1.01–1.25	325.311	0.171
Step 2	Depression	0.05 (0.00)	0.03–0.07	49.384	0.024
	Anxiety	0.03 (0.01)	0.00–0.05		
BD					
Step 1	BIDQ	1.29 (0.06)	1.15–1.42	354.562	0.184
Step 2	Depression	0.04 (0.01)	0.01–0.06	30.681	0.025
	Distress	0.03 (0.00)	0.01–0.05		
Bingeing					
Step 1	BIDQ	0.79 (0.04)	0.70–0.88	282.938	0.152
Step 2	Guilt-NBE	−0.12 (0.02)	−0.15–(−0.08)	33.536	0.070
Step 3	Anxiety	0.06 (0.00)	0.04–0.07	21.981	0.011
Purging					
Step 1	BIDQ	0.45 (0.03)	0.38–0.51	169.232	0.097
	Guilt-NBE	−0.13 (0.02)	−0.17–(−0.08)		
Step 2	Guilt-Repair	−0.01 (0.02)	−0.05–0.04	36.472	0.059
	Shame-NSE	−0.03 (0.02)	−0.07–0.02		
Step 3	Depression	0.01 (0.00)	0.01–0.03	47.080	0.071
	Anxiety	0.06 (0.00)	0.04–0.07		

Unstandardised *β* coefficients (SE) and 95% CI for independent variables are presented as covariate-adjusted, controlling for gender, location, and BMI. In the final model, all independent variables included were statistically significant. BD, body dissatisfaction; BIDQ, body image disturbance questionnaire; EDE-Q, eating disorder examination questionnaire; ER, eating restraint; GASP, guilt and shame proneness scale; Guilt-NBE, guilt-negative behavior evaluation; Shame-NSE, shame-negative self-evaluation; SWO, shape and weight over-evaluation.

### 3.5 Path analysis

**Figure 2:**
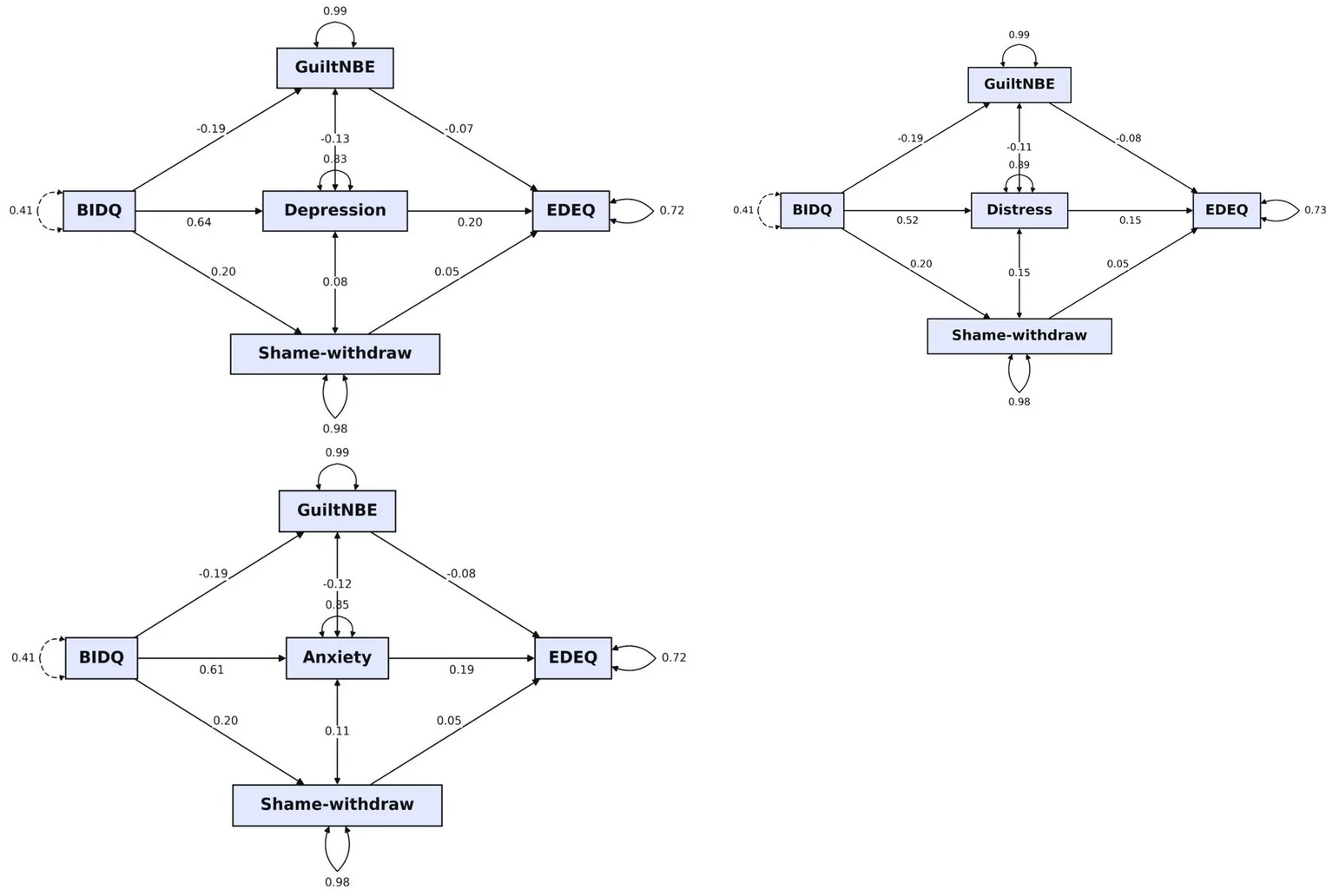
Mediating effects of depression, anxiety, and distress, together with the GASP subscales Guilt–NBE and Shame–Withdraw, on the relationship between body image disturbance and eating disorder symptoms. Separate path models are presented for each mediating variable; all path coefficients are standardized and statistically significant. BIDQ, Body Image Disturbance Questionnaire; GuiltNBE, Guilt–Negative Behavior Evaluation; EDEQ, Eating Disorder Examination Questionnaire.

Path analysis was conducted to examine the mediating roles of GASP sub-dimensions (Guilt-NBE and Shame-Withdraw) and mental health components (depression, anxiety, and distress) in the relationship between BIDQ and EDE-Q (Figure 2). All standardized *β* coefficients within the proposed models were found to be statistically significant (Figure 2, Supplementary Table S1). In the model incorporating depression as a mediator, the independent variable (BIDQ) and the mediating variables (depression, Guilt-NBE, and Shame-Withdraw) accounted for 28.3% of the total variance in eating behaviors (Supplementary Table S1). The analysis revealed that Guilt-NBE (indirect effect *β*[SE] = 0.02[0.00]), Shame-Withdraw (*β*[SE] = 0.01[0.00]), and depression (*β*[SE] = 0.13[0.01]) partially mediated the relationship between BIDQ and EDE-Q, yielding a total indirect effect of 0.15 (95% CI: [0.114–0.183], *z*-value = 8.491 and *p* < 0.001) (Supplementary Table S1). In the model evaluating anxiety, the explanatory power remained at 28.3%. While Guilt-NBE and anxiety significantly mediated the relationship between BIDQ and EDE-Q, the mediating effect of Shame-Withdraw was not statistically significant in this specific model. The strongest standardized path coefficients were observed from BIDQ to anxiety (*β*[SE] = 0.62[0.03]) and to EDE-Q (0.61[0.03]) (Supplementary Table S1). When distress was included as a mediator, the model explained 27.3% of the variance in EDE-Q scores, with Guilt-NBE and distress identified as significant mediators. The total effect of BIDQ on EDE-Q was found to be highest in the depression model (0.764), followed by the anxiety (0.722) and distress (0.704) models. Furthermore, the mediating variables accounted for 19.5% of the total effect in the depression model, 19.3% in the anxiety model, and 14.7% in the distress model (Figure 2, Supplementary Table S1).

## 4 Discussion

This study contributes to the growing body of literature examining the concurrent mediating roles of guilt, shame, depression, anxiety, and distress in the relationship between body image disturbance and eating disorder behaviors. The findings reveal a strong and significant relationship between the BIDQ and the EDE-Q; individuals at high risk for eating disorders showed a significant increase in depression, anxiety, and distress levels with moderate clinical effect sizes. Path analyses confirmed that Guilt-NBE, Shame-Withdrawal, and depression were consistent partial mediators in the BIDQ-EDE-Q relationship. Notably, anxiety emerged as a significant mediator of BIDQ and eating behaviors, explaining a significant portion of the total variance.

This finding is consistent with systematic reviews and meta-analyses examining the prevalence of eating disorders in large population samples, which report that lifetime prevalence is approximately 3.5 times higher in females than in males (females 2.58%; males 0.74%) (38). Previous research suggests that females are exposed to stronger sociocultural pressures related to the “thinness ideal,” which contributes to body dissatisfaction and the development of disordered eating behaviors (39). Therefore, the gender difference observed in our study may reflect the internalization of these sociocultural standards and their influence on eating attitudes.

In addition, individuals residing in metropolitan areas showed a significantly higher risk of eating disorder symptoms compared with those living in rural areas. This finding aligns with studies documenting the amplifying effect of industrialization and urbanization on eating disorder risk (40, 41). For example, the prevalence of bulimia nervosa in urban areas in the Netherlands has been reported to be approximately five times higher than in rural regions (41). Environmental factors associated with metropolitan life, such as Westernized beauty ideals, increased media exposure, and social comparison, may intensify body image concerns and promote maladaptive eating behaviors. Accordingly, modernization and urban lifestyles have been identified as important environmental contributors to eating disorder risk (40, 42).

Participants with higher eating disorder symptoms had significantly higher BIDQ scores, with a large effect size. In hierarchical regression analyses, BIDQ emerged as the strongest predictor of eating disorder symptoms, explaining 24% of the variance. This effect remained significant even after controlling for gender, location, and BMI, suggesting that body image disturbance is robustly and independently associated with eating pathology in this sample (43, 44). Although BMI differed between groups, the explanatory power of BIDQ remained independent of BMI, supporting previous findings that the overvaluation of body shape and weight predicts eating disorder severity regardless of BMI (43, 45). These findings indicate that body image disturbance represents a central component of eating pathology not only in clinically diagnosed individuals but also in at-risk groups within the general population (46).

Path analyses further revealed the mediating patterns of emotional variables in the relationship between BIDQ and EDE-Q. In the depression model, Guilt-NBE, Shame-Withdrawal, and depression acted as significant mediators, and the model explained 28.3% of the total variance. Similarly, previous studies have shown that anxiety and depression mediate the relationship between body image dissatisfaction and disordered eating behaviors (47, 48). These findings are consistent with the negative effect pathway proposed in the Dual-Pathway model, which suggests that body image disturbance contributes to bulimic pathology through depressive symptoms and restrictive eating behaviors (49). Network analyses of eating disorder psychopathology have also identified post-eating guilt and the overvaluation of body shape and weight as central symptoms linking body image disturbance with anxiety and depression (50, 51).

In the anxiety model, the explanatory power remained similar (28.3%), with mediating variables accounting for 19.3% of the total effect. The strong path coefficient between BIDQ and anxiety suggests that appearance-related psychological stress may be associated with higher anxiety, which in turn shows an indirect association with disordered eating behaviors. In contrast, the mediating effect of Shame-Withdrawal was not significant in this model, indicating that anxiety-related eating pathology may be more strongly influenced by emotion regulation processes rather than shame-based responses. In the stress model, the explanatory power slightly decreased to 27.3%, and mediators accounted for 14.7% of the total effect. While Guilt-NBE and stress emerged as significant mediators, Shame-Withdrawal did not reach statistical significance. This pattern suggests that eating disorder psychopathology may be more strongly influenced by the ruminative and evaluative cognitive processes associated with depression and anxiety than by the physiological arousal associated with stress (52).

The consistent negative association between Guilt-NBE and EDE-Q scores supports the interpretation that this dimension reflects adaptive guilt. Behavior-focused guilt may be linked to greater self-regulation, potentially by directing attention toward specific actions.

Neurologically, guilt processing is mediated by a network of brain regions, such as the anterior cingulate cortex and the medial prefrontal cortex, particularly the dorsal medial prefrontal cortex (53).

In contrast, shame involves broader self-criticism and has been linked to the maintenance of eating disorder pathology (13, 14). Accordingly, lower Guilt-NBE scores were associated with self-regulatory difficulties and higher levels of eating disorder symptoms, a pattern consistent with the conceptualisation of behavior-focused guilt as an adaptive, self-regulatory emotional process (54).

Regarding the Shame-Withdrawal dimension, individuals with higher eating disorder symptoms showed significantly higher levels of withdrawal-related shame. However, when BIDQ, Guilt-NBE, and affective variables were simultaneously included in the model, the independent predictive effect of Shame-Withdrawal weakened and remained significant only in the depression model. This pattern suggests that the association between withdrawal-related shame and eating disorder symptoms may largely reflect its co-occurrence with depressive affect. This finding is consistent with the interpersonal theory of shame, which conceptualises shame as a fundamentally social emotion associated with perceived inferiority, subordination, and the anticipation of rejection or exclusion by others (55–57). shame-induced withdrawal may be conceptually understood as a defensive strategy associated with the avoidance of perceived social threat. Within this framework, withdrawal-related shame, depressive affect, and disordered eating behaviors may co-occur as part of an interrelated symptom cluster, although the present cross-sectional design does not permit inferences regarding the temporal sequence or directionality of these associations (58). This interpretation is further supported by compassion-focused therapy models, which identify shame-based self-criticism and social disengagement as central maintaining factors in eating pathology (59, 60). The context-dependent nature of Shame-Withdrawal as a mediator significant in the depression model but not in anxiety or stress models suggests that its influence on eating pathology may be primarily channeled through rumination and mood-congruent cognitive processes rather than acute physiological arousal (61, 62). This dynamic suggests that shame-related responses may be associated with disengagement from self-regulation rather than with active modulation of eating behaviors (63).

These findings are particularly noteworthy in the Turkish sociocultural context, where body image concerns and disordered eating behaviors have received comparatively limited empirical attention relative to Western populations. Turkey occupies a unique cultural position at the intersection of Eastern and Western influences, characterized by increasing exposure to Westernized beauty ideals through social media and urbanization, alongside deeply embedded cultural norms regarding body shape, femininity, and emotional expression (64). In this context, the strong association between body image disturbance and eating pathology observed in the present study mirrors findings reported in Western literature, suggesting that the psychological mechanisms linking body dissatisfaction to disordered eating may transcend cultural boundaries. However, the specific emotional pathways through which body image disturbance operates particularly the roles of guilt and shame may be shaped by culturally specific patterns of self-evaluation and interpersonal concern (65). Shame, in particular, carries a distinct social and relational weight in Turkish culture, where social reputation, honor, and collective judgment are salient determinants of self-worth (64, 65). The replication of shame- and guilt-based mediation pathways in this non-Western sample therefore extends the cross-cultural generalisability of existing theoretical models and underscores the relevance of culturally informed approaches to eating disorder research and intervention in Turkey.

This need for cultural sensitivity carries direct implications for clinical practice. In Turkey, dietitians are frequently the first and sometimes the only health professionals consulted by individuals with body image concerns or disordered eating patterns. This position confers a distinctive responsibility for early identification. Equipping dietitians with the competence to recognize the early signs of body image disturbance and shame-based eating behavior, alongside standard nutritional counseling, may therefore represent a critical opportunity for early intervention. Direct evidence supports this need. In a recent study of Turkish dietitians, fat phobia was identified in 66.9% of participants, and weight bias was associated with more negative appraisals of a patient’s nutritional and biochemical status even when the reason for consultation was unrelated to body weight (66). This suggests that bias awareness and a non-judgmental approach are as decisive as nutritional knowledge in dietetic practice. A comparable training gap has been documented among psychiatry residents in Turkey, most of whom reported inadequate training and a lack of clinical supervision in eating disorders (67). Whether a similar gap exists within Turkish dietetics curricula remains, to our knowledge, empirically unexamined and warrants dedicated investigation.

The emergence of the BIDQ as the strongest predictor of eating disorder symptoms provides a concrete basis for practice-oriented recommendations. Integrating structured body image screening tools and foundational training in motivational, non-judgmental counseling into dietetics curricula may enable dietitians to identify at-risk individuals earlier and to refer them appropriately to mental health services, rather than addressing body dissatisfaction through dietary restrictions alone. Indeed, a purely restrictive approach has been suggested to sustain rather than alleviate body image-related distress. Finally, a systematic review of cognitive-behavioral family therapy reported that family-inclusive interventions were associated with more durable improvements than individual treatment in eating disorder populations (68). In the dietetic context, family-based counselling incorporating a nutritional component may address the relational and shame-based dynamics identified here more effectively than individual counselling alone, particularly for adolescents and young adults.

### 4.1 Strengths and limitations

The study’s large sample size (*n* = 1,576), use of culturally validated Turkish instruments (EDE-Q-13, BIDQ, GASP, DASS-21), and diverse recruitment across several geographical areas are strengths that enhance the generalizability and reliability of the findings. The use of multiple complementary statistical approaches strengthened methodological rigor by employing hierarchical regression and path analysis, with effect sizes reported and key covariates, such as gender, BMI, and location, controlled for. Notably, the path analysis model strengthened the study by moving from simple associations to empirically testing the mediation pathways, providing new insight into the mechanisms by which depression, anxiety, distress, and guilt/shame partially mediate the relationship between body image disturbance and eating disorders.

Despite these strengths, several limitations should be considered when interpreting the findings. First, the cross-sectional design precludes causal inferences about the relationships among body image disturbance, emotional factors, and disordered eating behaviors. Longitudinal studies are therefore required to clarify the temporal direction of these associations. Second, participants were recruited via social media platforms using a snowball sampling strategy, a method that introduces several interrelated sources of sampling bias warranting systematic consideration. This recruitment approach likely resulted in the overrepresentation of young, digitally active, and urban-dwelling individuals demographic strata in which disordered eating attitudes and appearance-related concerns are documented at elevated rates relative to the general population. Consequently, the prevalence estimates derived from the present sample, particularly high-risk.

EDE-Q profiles, may overestimate the true population rate and fail to accurately represent the distribution of eating pathology across the broader Turkish adult population. Furthermore, the observed gender imbalance, with women comprising 61% of the sample, may have systematically inflated the estimated effect sizes of the guilt- and shame-based mediation pathways identified in the path analysis. Shame-proneness and its behavioral manifestations, including withdrawal and concealment tendencies, are consistently more pronounced among women, and the indirect effects of Guilt-NBE and Shame-Withdraw may therefore partly reflect a sex-weighted artifact rather than a population-invariant psychological mechanism. Whether these mediation pathways operate with equivalent magnitude across gender groups remains unexamined in the present study, and measurement invariance testing across gender is recommended in future investigations. Finally, the systematic underrepresentation of rural populations constitutes an additional constraint on external validity. Rural-dwelling individuals in Turkey differ from their urban counterparts across several dimensions relevant to the present study, including socioeconomic status, exposure to idealized body image standards, and culturally mediated patterns of emotional expression, each of which may moderate the associations among body image disturbance, shame-proneness, and disordered eating. Future research employing stratified probability sampling with explicit urban–rural and age-cohort stratification is therefore necessary to establish population-representative prevalence estimates and to evaluate the replicability of the identified mediation structure across diverse sociocultural contexts within Turkey.

Third, all variables were assessed using self-report instruments. Self-reported anthropometric data, including height and weight, may be subject to reporting inaccuracies, and responses related to eating behaviors and emotional experiences may have been influenced by social desirability bias. Additionally, the use of a median split for EDE-Q scores, although useful for statistical group comparisons, differs from clinically established cut-off values and may limit direct comparability with clinical studies. Similarly, the GASP subscales used in the mediation models were chosen based on their observed bivariate associations with eating pathology rather than a pre-specified theoretical model. Thus, the identified mediation structure should be regarded as exploratory and requires confirmation in independent samples using theoretically pre-defined mediators. Finally, the sample was predominantly composed of young adults from Eastern Anatolia, and cultural differences in body ideals, emotional expression, and eating attitudes may limit the generalizability of the findings to other sociocultural contexts. Future research using longitudinal designs, more diverse and representative samples, and objective anthropometric measurements would help to further clarify the mechanisms underlying the relationship between body image disturbance and disordered eating behaviors.

## 5 Conclusion

The findings of this study identify body image disturbance as a central predictor of eating disorder behaviors, whose association appears to operate through a group of emotional and psychological mediators. Guilt oriented toward negative behavioral evaluation may confer a degree of self-regulatory protection, whereas shame-driven withdrawal and affective distress, most notably anxiety and depression, appear to be associated with greater susceptibility to disordered eating. Taken together, these results suggest that symptom-focused approaches alone may be insufficient in eating disorder interventions. Additionally, the emotional architecture underlying body image disturbance warrants targeted clinical attention. Accordingly, comprehensive interventions targeting maladaptive shame, anxiety, and depressive symptoms, along with efforts to reinforce adaptive guilt processing, may constitute meaningful therapeutic targets. Longitudinal research designs are warranted to further elucidate the mechanisms through which body image disturbance contributes to eating pathology across demographically diverse populations.

## Data Availability

The data analyzed in this study is subject to the following licenses/restrictions: the data presented in this study are available on request from the corresponding author due to privacy restrictions. Requests to access these datasets should be directed to nazlinuraslan@ktu.edu.tr.

## Footnotes

1
https://jasp-stats.org
