# Supplementary material for: The mediating role of psychological factors in the relationship between body image dissatisfaction and eating disorders: a cross-sectional study in adults
Source: Front Nutr. 2026 Aug 24;13:1881290. doi: 10.3389/fnut.2026.1881290 (PMC13547021; doi:10.3389/fnut.2026.1881290)
Supplement: Supplementary file 1 [file Supplementary_file_1.docx]

**Figure S1.** Pearson correlation heatmap matrix of EDE-Q subscales with BIDQ, GASP components, and mental health indicators

**Table S1.** Estimated coefficients and summary statistics of path analyses for depression, anxiety, and distress models

|  | | | Estimate | Std. Error | z-value | p | 95% CI | |
| --- | --- | --- | --- | --- | --- | --- | --- | --- |
|  |  |  |  |  |  |  | **Lower** | **Upper** |
| For Depression | | | | | | | | |
| Guilt-NBE | → | EDE-Q | -0.074 | 0.024 | -3.159 | 0.002 | -0.120 | -0.028 |
| Depression | → | EDE-Q | 0.195 | 0.024 | 8.172 | < .001 | 0.149 | 0.242 |
| Shame-Withdraw | → | EDE-Q | 0.053 | 0.023 | 2.252 | 0.024 | 0.007 | 0.098 |
| BIDQ | → | EDE-Q | 0.615 | 0.037 | 16.775 | < .001 | 0.543 | 0.687 |
| BIDQ | → | Guilt-NBE | -0.186 | 0.039 | -4.768 | < .001 | -0.262 | -0.109 |
| BIDQ | → | Depression | 0.638 | 0.036 | 17.831 | < .001 | 0.568 | 0.708 |
| BIDQ | → | Shame-Withdraw | 0.196 | 0.039 | 5.042 | < .001 | 0.120 | 0.272 |
| For Anxiety | | | | | | | | |
| Guilt-NBE | → | EDE-Q | -0.076 | 0.023 | -3.225 | 0.001 | -0.122 | -0.030 |
| Anxiety | → | EDE-Q | 0.192 | 0.024 | 8.087 | < .001 | 0.146 | 0.239 |
| Shame-Withdraw | → | EDE-Q | 0.047 | 0.023 | 2.022 | 0.043 | 0.001 | 0.093 |
| BIDQ | → | EDE-Q | 0.623 | 0.036 | 17.140 | < .001 | 0.552 | 0.695 |
| BIDQ | → | Guilt-NBE | -0.186 | 0.039 | -4.768 | < .001 | -0.262 | -0.109 |
| BIDQ | → | Anxiety | 0.610 | 0.036 | 16.898 | < .001 | 0.539 | 0.681 |
| BIDQ | → | Shame-Withdraw | 0.196 | 0.039 | 5.042 | < .001 | 0.120 | 0.272 |
| For Distress | | | | | | | | |
| Guilt-NBE | → | EDE-Q | -0.082 | 0.024 | -3.467 | < .001 | -0.128 | -0.036 |
| Distress | → | EDE-Q | 0.154 | 0.023 | 6.536 | < .001 | 0.108 | 0.200 |
| Shame-Withdraw | → | EDE-Q | 0.048 | 0.024 | 1.999 | 0.046 | 9.194×10^-4^ | 0.094 |
| BIDQ | → | EDE-Q | 0.660 | 0.036 | 18.428 | < .001 | 0.589 | 0.730 |
| BIDQ | → | Guilt-NBE | -0.186 | 0.039 | -4.768 | < .001 | -0.262 | -0.109 |
| BIDQ | → | Distress | 0.520 | 0.037 | 14.048 | < .001 | 0.447 | 0.592 |
| BIDQ | → | Shame-Withdraw | 0.196 | 0.039 | 5.042 | < .001 | 0.120 | 0.272 |

Delta method standard errors, normal theory confidence intervals, ML estimator.

**Table S2.** Direct and total effects of the relationship between BIDQ and EDE-Q across depression, anxiety, and distress models

|  |  | |  | Estimate | Std. Error | z-value | p | 95% CI | |
| --- | --- | --- | --- | --- | --- | --- | --- | --- | --- |
|  |  |  |  |  |  |  |  | **Lower** | **Upper** |
| BIDQ→ EDE-Q | | **For Depression** | | | | | | | |
|  |  | Direct | | 0.615 | 0.037 | 16.775 | <.001 | 0.543 | 0.687 |
|  |  | Total | | 0.764 | 0.034 | 22.347 | <.001 | 0.697 | 0.831 |
|  |  | **For Anxiety** | | | | | | | |
|  |  | Direct | | 0.623 | 0.036 | 17.140 | <.001 | 0.552 | 0.695 |
|  |  | Total | | 0.722 | 0.031 | 22.218 | <.001 | 0.689 | 0.781 |
|  |  | **For Distress** | | | | | | | |
|  |  | Direct | | 0.660 | 0.033 | 18.428 | <.001 | 0.589 | 0.730 |
|  |  | Total | | 0.704 | 0.034 | 21.962 | <.001 | 0.657 | 0.793 |

Delta method standard errors, normal theory confidence intervals, ML estimator.

**Table S3.** Summary statistics of indirect effects in the path analyses

| Independet | Mediators | | | Dependent | Estimate | SE | z-value | p | 95% CI | |  |
| --- | --- | --- | --- | --- | --- | --- | --- | --- | --- | --- | --- |
|  |  |  |  |  |  |  |  |  | **Lower** | **Upper** |  |
| For Depression | | | | | | | | | | | |
| BIDQ | → | Guilt-NBE | → | EDE-Q | 0.014 | 0.005 | 2.634 | 0.008 | 0.004 | 0.024 |  |
|  | → | Depression | → |  | 0.125 | 0.017 | 7.429 | < .001 | 0.092 | 0.158 |  |
|  | → | Shame-Withdraw | → |  | 0.010 | 0.005 | 2.056 | 0.040 | 4.8×10^-4^ | 0.020 |  |
| BIDQ | → | | | EDE-Q | 0.149 | 0.018 | 8.491 | < .001 | 0.114 | 0.183 |  |
| For Anxiety | | | | | | | | | | | |
| BIDQ | → | Guilt-NBE | → | EDE-Q | 0.014 | 0.005 | 2.671 | 0.008 | 0.004 | 0.024 |  |
|  | → | Anxiety | → |  | 0.117 | 0.016 | 7.294 | < .001 | 0.086 | 0.149 |  |
|  | → | Shame-Withdraw | → |  | 0.009 | 0.005 | 1.877 | 0.061 | -4.1×10^-4^ | 0.019 |  |
| BIDQ | → | | | EDE-Q | 0.141 | 0.017 | 8.356 | < .001 | 0.108 | 0.174 |  |
| For Distress | | | | | | | | | | |  |
| BIDQ | → | Guilt-NBE | → | EDE-Q | 0.015 | 0.005 | 2.804 | 0.005 | 0.005 | 0.026 |  |
|  | → | Distress | → |  | 0.080 | 0.013 | 5.926 | < .001 | 0.053 | 0.106 |  |
|  | → | Shame-Withdraw | → |  | 0.009 | 0.005 | 1.858 | 0.063 | -5.1×10^-4^ | 0.019 |  |
| BIDQ | → | | | EDE-Q | 0.104 | 0.015 | 7.194 | < .001 | 0.076 | 0.133 |  |

Delta method standard errors, normal theory confidence intervals, ML estimator.

**Table S4.** Coefficients of determination (R²) for latent variables in the path analyses.

| Dependent variable | R² | Dependent variable | R² | Dependent variable | R² |
| --- | --- | --- | --- | --- | --- |
| For Depression | | **For Anxiety** | | **For Distress** | |
| EDE-Q | 0.283 | EDE-Q | 0.283 | EDE-Q | 0.273 |
| Guilt-NBE | 0.014 | Guilt-NBE | 0.014 | Guilt-NBE | 0.014 |
| Depression | 0.168 | Anxiety | 0.153 | Distress | 0.111 |
| Shame-Withdraw | 0.016 | Shame-Withdraw | 0.016 | Shame-Withdraw | 0.016 |
